# Fear of cancer recurrence as a pathway from fatigue to psychological distress in mothers who are breast cancer survivors

**DOI:** 10.1002/smi.3180

**Published:** 2022-07-08

**Authors:** Carissa Nadia Kuswanto, Jessica Sharp, Lesley Stafford, Penelope Schofield

**Affiliations:** ^1^ Department of Psychological Sciences Swinburne University of Technology Hawthorn Victoria Australia; ^2^ Women's Mental Health Team The Royal Women's Hospital Melbourne Victoria Australia; ^3^ Melbourne School of Psychological Sciences The University of Melbourne Melbourne Victoria Australia; ^4^ Iverson Health Innovation Research Institute Swinburne University of Technology Hawthorn Victoria Australia; ^5^ Behavioural Sciences Unit Health Services Research and Implementation Sciences Peter MacCallum Cancer Centre Melbourne Victoria Australia; ^6^ Sir Peter MacCallum Department of Oncology The University of Melbourne Melbourne Victoria Australia

**Keywords:** anxiety, breast cancer survivors, depression, fatigue, fear of cancer recurrence, mothers, psychological distress

## Abstract

Fatigue is prevalent and pervasive among breast cancer survivors. Mothers are particularly susceptible to fatigue due to the ongoing demands of their caring role. While fatigue has been associated with psychological distress in prior research, the pathway by which fatigue translates into psychological distress is unclear. Given the theoretical and empirical links between fatigue, fear of cancer recurrence (FCR) and psychological distress, the role of FCR in mediating the relationship between fatigue and psychological distress in mothers who are breast cancer survivors was investigated. Ninety‐two mothers who were breast cancer survivors completed the Depression, Anxiety and Stress Scale, PROMIS‐Cancer Fatigue Short Form and Concerns About Cancer Recurrence scale in an online survey. Mediation analysis via PROCESS was used to examine whether fatigue predicted depression, anxiety or stress through FCR. Fear of cancer recurrence mediated the relationships between fatigue and anxiety and fatigue and stress, while fatigue directly predicted depression. This study highlights FCR as a potential pathway to anxiety and stress in response to ongoing fatigue, and as a mechanism of action to reduce psychological distress among mothers who are breast cancer survivors. Future research examining this pathway from fatigue to psychological distress should also explore the nature of mothers' fears about their cancer recurring.

## 1 INTRODUCTION

Cancer‐related fatigue is a distressing, persistent, subjective sense of physical, emotional and/or cognitive tiredness or exhaustion related to cancer or cancer treatment, which often interferes with day‐to‐day functioning (Hilarius et al., 2011; Stone & Minton, 2008; Yavuzsen et al., 2009). Fatigue is reported as the greatest contributor to symptom burden in mothers with cancer 1 year after cancer therapy has been completed (Gotze et al., 2015), and may continue to be prevalent 5–10 years after cancer diagnosis (Bower et al., 2006; Hofman et al., 2007). Mothers are often presented with the struggle of balancing their family's needs and their own needs, in addition to the perceived societal demands to resume parenting responsibility and housekeeping despite their illness (Fisher & O’Connor, 2012; Kuswanto et al., 2018; Semple & McCance, 2010). Arguably these demands make mothers in particular more vulnerable to high levels of fatigue. Physical limitations due to cancer such as fatigue can also greatly interfere with mothers' daily parenting activities (Gotze et al., 2015; Hofman et al., 2007; Khoshknabi & Davis, 2006; Mackenzie, 2014) and their perceived ability to fulfil parenting demands (Kuswanto et al., 2018; Tavares et al., 2017), with the effects also felt by their caregivers and family members (Hofman et al., 2007).

Fatigue has also been consistently linked to mood disturbance and psychological distress, such as anxiety and depression, among cancer patients and survivors (Costanzo et al., 2007; Hofman et al., 2007; Smets et al., 1998; Stone et al., 2000; Stone & Minton, 2008). Nevertheless, the pathway by which fatigue translates into psychological distress is unclear (Hofman et al., 2007; Smets et al., 1998). A mounting body of research highlights a strong link between cancer survivors' physical symptoms, including fatigue, and their fears about cancer recurring (Crist & Grunfeld, 2013; Kelada et al., 2019; Lebel et al., 2018; Simard et al., 2013), indicating that cancer survivors may interpret their fatigue as a possible sign of cancer recurrence or progression. Accordingly, a case can be made for heightened fear of cancer recurrence (FCR) as a possible mechanism in the pathway from fatigue to psychological distress.

Leventhal's Common Sense Model (CSM; Lebel et al., 2018; Lee‐Jones et al., 1997), suggests that the perception of internal cues (e.g., physical symptoms and fatigue) may trigger anxious preoccupation and rumination about cancer recurring and may manifest in repetitive checking behaviour and seeking reassurance from family, doctors and other healthcare practitioners (Lebel et al., 2018; Lee‐Jones et al., 1997; McGinty et al., 2016; Vickberg, 2003). The CSM illustrates how somatic experiences that represent cancer may trigger a series of cognitive processes, whereby somatic symptoms such as pain and fatigue are interpreted as symptoms of cancer recurring. In addition, Mishel's Uncertainty in Illness theory proposes that the uncertain nature of cancer may heighten FCR (Lebel et al., 2018; Mishel, 1988). Under conditions of uncertainty and unpredictability, somatic triggers such as pain and fatigue are more likely to be interpreted as signs of recurrence, which in turn contribute to FCR (Lebel et al., 2018). Both theories postulate subsequent emotional processes, for example, anxiety or distress, in response to such interpretations of somatic experiences (Lee‐Jones et al., 1997). A more comprehensive, contemporary cognitive processing model of FCR (Fardell et al., 2016) also recognises the centrality of self‐focussed sensitivity or hypervigilance to signs that cancer may have returned, as well as the links between FCR and distress such as anxiety or depression.

Accordingly, FCR is one of the strongest predictors of psychological distress symptoms in long term cancer survivors (Deimling et al., 2006; Koch et al., 2014). Furthermore, cancer survivors with at least one child also report higher FCR than those without children (Arès et al., 2014; Koch et al., 2013; Kuswanto et al., 2018; Lebel et al., 2012; Mehnert et al., 2009; Tavares et al., 2017; Wan et al., 2018), which is consistent with the cognitive processing model of FCR that identifies life circumstances, such as the demands of a caring role, as a vulnerability factor for heightened FCR (Fardell et al., 2016). Parents, particularly mothers, who have previously been diagnosed with cancer are also likely to worry about the negative impact that cancer recurrence would have on their children's physical and psychological wellbeing (Arès et al., 2014; Kuswanto et al., 2018; Tavares et al., 2017; Visser et al., 2005; Wan et al., 2018). It is thus not surprising that FCR has been associated with higher psychological distress in mothers who are breast cancer survivors (Arès et al., 2014; Wan et al., 2018).

Taken together, it is therefore plausible that fatigue provokes FCR in cancer survivors, and FCR is in turn associated with symptoms of psychological distress; indicating the potential role of FCR in mediating the relationship between fatigue and psychological distress. Moreover, a recent study demonstrated FCR mediated between fatigue and health outcomes among haematological cancer survivors, representing a pathway from fatigue to physical functioning and quality of life (Esser et al., 2019). These findings highlight the possible clinical benefits of targeting FCR to minimise the detrimental effects of fatigue on cancer survivors' quality of life without needing to directly reduce fatigue. Despite the potential clinical benefits, the associations between fatigue, FCR and psychological distress have not yet been explored in mothers who are breast cancer survivors, even though fatigue poses considerable parenting and psychological issues.

**FIGURE 1:**
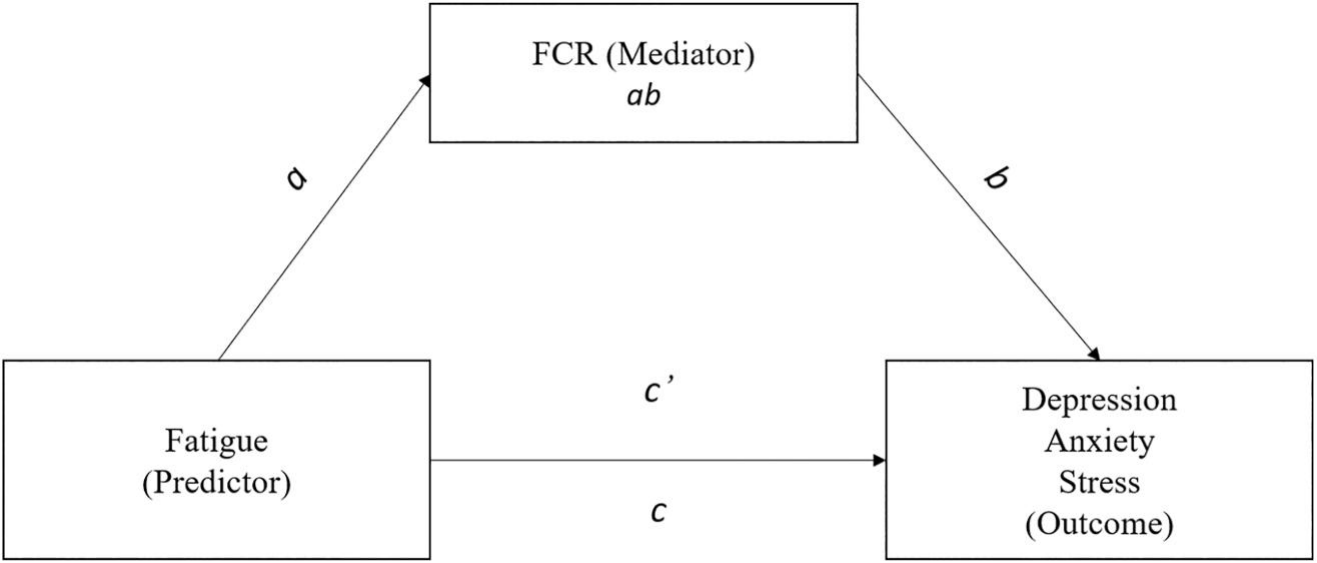
A graphical and statistical representation of the mediation model. FCR, fear of cancer recurrence

In view of the potential clinical benefits of better understanding psychological distress in mothers who are breast cancer survivors, it is pertinent to examine the complex relationship between fatigue, FCR and psychological distress. The current study therefore investigated the extent to which FCR mediates the relationship between fatigue and symptoms of psychological distress among mothers who are breast cancer survivors. It was hypothesised that fatigue would be associated with symptoms of psychological distress both directly, and indirectly via FCR, in our sample of mothers who are breast cancer survivors (Figure 1). Acknowledging that depression, anxiety and stress are distinct symptoms of psychological distress (Lovibond, 1998; Oathes et al., 2015; Stafford et al., 2016), the current study examined the mediating role of FCR for depression, anxiety and stress separately.

## 2 METHOD

### 2.1 Participants

Mothers were recruited from the Breast Cancer Network Australia (BCNA); an online network providing information and support for women affected by breast cancer in Australia. The inclusion criteria were being 18 years or older, a mother at the time of receiving the diagnosis, being able to give informed consent and to complete the online survey in English. Ethics approval was obtained from Swinburne University Human Research Ethics Committee (SHR Project 2017/008).

### 2.2 Measures

#### 2.2.1 Demographic and clinical information

Participants completed questions about demographics including age, relationship status, number and ages of children, highest education level, employment status (i.e., not working or working full‐time/part‐time); and clinical details including known disease stage, type of treatment received, and time (months) since receiving the cancer diagnosis and last treatment.

#### 2.2.2 Depression, anxiety, and stress scale (DASS‐21)

The DASS‐21 is a self‐report questionnaire assessing three dimensions of negative emotional states: depression, anxiety and stress (Lovibond & Lovibond, 1995; Page et al., 2007). There are seven items that measure each dimension, and each item is measured using a 4‐point Likert‐type scale, ranging from 0 (did not apply to me at all) to 3 (applied to me very much, or most of the time). Scores of at least 10 for depression, 8 for anxiety, and 15 for stress indicate mild to extremely severe distress for each subscale. The Depression, Anxiety and stress scale (DASS) full‐scale scores are calculated by multiplying the DASS‐21 subscale scores by two in accordance with guidelines provided by Lovibond and Lovibond (1995). For our study, the internal reliability for each subscale was high (*α* = 0.91 for depression; *α* = 0.81 for anxiety; *α* = 0.86 for stress).

#### 2.2.3 Patient reported outcome measurement information System's (PROMIS) Cancer Fatigue Short Form (PROMIS‐CFSF)

Cancer related fatigue was assessed with the PROMIS‐CFSF, a reliable and valid measure of fatigue in cancer populations (Cessna et al., 2016). It has seven items measuring the frequency of fatigue in the past 7 days. Each item is scored on a 5‐point Likert scale, ranging from 1 (never) to 5 (always). After reverse scoring item 7, a higher sum of scores indicates greater fatigue. The internal reliability in our study was high (*α* = 0.81).

#### 2.2.4 Concerns about recurrence scale (CARS)

Fear of cancer recurrence was measured by the first four items of the Concerns about recurrence scale (CARS) (Vickberg, 2003), which assess the frequency, intensity and consistency of FCR, and potential for emotional distress caused by FCR, in the past month. Each item is scored on a Likert scale from 1 (not at all) to 6 (all the time). Higher scores suggest greater FCR, with scores rounded to three or four indicating moderate levels and scores rounded to five or six indicating high levels of FCR (Liu et al., 2011; Vickberg, 2003). The CARS has been used in past studies with breast cancer survivors (Dumalaon‐Canaria et al., 2016; Lebel et al., 2012; Liu et al., 2011; Lucas, 2013; van den Beuken‐van Everdingen et al., 2008). For our study, the internal reliability was high (*α* = 0.93).

### 2.3 Design and procedure

The study was cross‐sectional with a convenience sample of volunteers from the BCNA. After providing consent, participants were requested to anonymously complete the measures online through Qualtrics software (Qualtrics, Provo, UT, USA), a secure web‐based tool to conduct online surveys. The survey took participants approximately 30–45 min to complete.

### 2.4 Data analysis

Power analysis was performed using G*Power 3.1 (Faul et al., 2007). Based on a minimum sample size required to detect moderate effects for a multiple regression model with 3 predictors (effect size = 0.15, power level = 0.8, *p*‐value = 0.05), the minimum sample size required is 77 participants. Using Fritz and MacKinnon's (2007) approach to estimating power for bias‐corrected bootstrap tests of mediation, the estimated sample size needed for power level = 0.8 with medium effects indicated 71 participants.

Descriptive and correlational analyses were conducted using Statistical Package for Social Sciences Statistics (Version 25). Pearson's bivariate correlational analyses were conducted between the demographic and clinical factors, fatigue (PROMIS‐CFSF), FCR (CARS), and symptoms of psychological distress (depression, anxiety, and stress). Those with significant correlations (*p* < 0.05) with depression, anxiety or stress were included as covariates in the mediation analyses.

The mediation model with fatigue as the predictor, depression, anxiety and stress as the outcome variables, and FCR as the mediator was tested using PROCESS macro (Version 3.4) for SPSS Model 4 (Hayes, 2017). PROCESS Model 4 tests the effect of the predictor (fatigue) on potential mediator (FCR; the *a* path), the effect of the mediator on the outcome variable (depression, anxiety or stress; the *b* path), the total effect of the predictor on the outcome variable (the *c* path) and the direct effect of the predictor on the outcome variable with the mediator in the model (path *c*’) (Figure 1). A nonparametric bootstrapping method with 5000 bootstrap estimates was used to estimate the indirect effect (*ab*) of the independent variable on the dependent variable through the mediating variable (Hayes, 2017). The indirect effect is considered significantly different from zero when zero is not included within the 95% confidence interval (CI), such that the CI is consistently either positive or negative. If paths *a*, *b*, and *c* are significant and *c*’ is reduced compared to *c*, the criteria for partial mediation are met; if paths *a*, *b*, and *c* are significant and *c*’ becomes non‐significant, criteria for full mediation are met. The effect size was measured by the ratio of the indirect effect to the direct effect (Esser et al., 2019). The mediation analyses were conducted separately for depression, anxiety and stress.

## 3 RESULTS

### 3.1 Sample characteristics

A total of 146 participants provided informed consent to participate in the online survey. Thirteen participants consented but provided no data, and data from 41 participants were excluded due to incompleteness. In total, 92 mothers were included in the study. Table 1 summarises the characteristics of the sample and the descriptive statistics for the measures.

**TABLE 1:** Sample Demographic and clinical characteristics (*N* = 92)

Demographic and clinical variables	N	Mean	SD	Percentage (Range)
Age of participants	92	51.00	6.76	(34–69 years)
Age of the oldest child at survey	‐	21.30	9.09	(7–49 years)
Age of the youngest child at survey	‐	17.52	7.95	(4–46 years)
Number of children:				
1‐2	65	‐	‐	70.7%
≥ 3	27	‐	‐	29.3%
Relationship status:				
Without a partner	11	‐	‐	12.0%
With a partner	81	‐	‐	88.0%
Working full‐time/part‐time	72	‐	‐	78.3%
Completing tertiary education	80	‐	‐	87.0%
Living arrangement:				
With spouse/partner and children	66	‐	‐	71.7%
With children only	6	‐	‐	5.4%
Born outside of Australia	16	‐	‐	17.4%
Stage of cancer:				
Stage I	45	‐	‐	48.9%
Stage II	36	‐	‐	39.1%
Stage III‐IV/Unknown	11	‐	‐	12.0%
Time duration (in months) since diagnosis	‐	80.12	59.84	(13–351 months)
Time duration (in months) since surgery	‐	69.03	55.56	(5–348 months)
Treatment received				
Surgery/mastectomy	96	‐	‐	100%
Chemotherapy	68	‐	‐	73.9%
Radiotherapy	61	‐	‐	66.3%
Hormonal therapy	68	‐	‐	73.9%

Measures		Mean	SD	Possible range (observed range)
Depression, Anxiety and stress scale (DASS)				
Depression		6.48	7.98	0–42 (0–38)
Anxiety		4.30	6.03	0–42 (0–28)
Stress		10.91	8.11	0–42 (0–32)
PROMIS cancer fatigue short form (CFSF)		17.68	5.05	7–35 (8–28)
Concern about cancer recurrence (CARS)		3.75	1.34	1–6 (1–6)

Abbreviation: FCR, fear of cancer recurrence.

The DASS subscale means for the sample were within the normal ranges for each symptom, though individual DASS subscale scores varied across participants from nil to extremely severe levels of distress. Notable percentages of participants (Depression: 26.1%, Anxiety: 17.4% and Stress: 27.2%) reported higher than normal levels of distress, from mild to extremely severe. The average PROMIS‐CFSF score was moderate in our sample (mean = 17.68, on possible range of 7–35), though 31.5% of the participants reported moderate‐to‐high levels of fatigue. Also, the average CARS score in our sample was moderate (mean = 3.75, SD = 1.34) on a 1–6 scale. However, more than half of the participants (68.5%) reported moderate‐to‐high FCR (range 3.0–6.0); 45.7% of participants reported moderate levels and 22.8% of participants reported high levels of FCR.

### 3.2 Correlations

Depression, anxiety and stress were significantly positively correlated with fatigue and FCR (Table 2). Fatigue, too, was significantly positively correlated with FCR. Depression and stress were not correlated with the demographic or clinical factors, but anxiety was positively correlated with age of mothers (*r* = 0.21, *p* = 0.046), age of the youngest child (*r* = 0.32, *p* < 0.01) and number of children (*r* = 0.24, *p* = 0.02); while being employed (full or part‐time) was negatively correlated with anxiety (*r* = −0.24, *p* = 0.02), indicating these variables as covariates for the mediation analyses predicting anxiety. There were no correlations between DASS subscales and relationship status, education status, stage of cancer and time since diagnosis or surgery. There were significant correlations between fatigue and time since surgery (*r* = −0.30, *p* < 0.01), and between FCR and time since surgery (*r* = −0.24, *p* = 0.02). The DASS subscales were moderately correlated, indicating that depression, anxiety and stress are related and yet distinct dimensions of psychological distress.

**TABLE 2:** Correlations between variables of interest

Variables	2	3	4	5	6	7	8	9
1. Depression	0.50**	0.56**	0.47**	0.35**	0.43**	0.10	0.19	−0.54
2. Anxiety		0.67**	0.39**	0.38**	0.21*	0.32**	0.24*	−0.24*
3. Stress			0.48**	0.42**	0.14	0.18	0.05	−0.16
4. Fatigue				0.43**	−0.22*	−0.16	0.08	−0.08
5. FCR					−0.09	−0.004	0.01	−0.12
6. Age of mothers						0.73**	0.08	−0.19
7. Age of youngest child							−0.001	−0.11
8. Number of children								−0.14
9. Employment status								

*Note*: Employment status: 0 = not working, 1 = working FT.

Abbreviations: FCR, fear of cancer recurrence; FT, Full time; PT, part time.

*p < 0.05; **p < 0.01 level (two‐tailed).

### 3.3 Mediation analyses

The mediation model predicted the indirect effect of fatigue through FCR, as well as the direct effect of fatigue on the psychological distress variables. Table 3 summarises the results of the mediation analyses for depression, anxiety and stress. For all models, the *c* paths that demonstrate the relationship between fatigue and depression, anxiety and stress were significant and positive, indicating that fatigue predicted symptoms of psychological distress. The *a* paths, representing the relationships between fatigue and FCR, were also all significant and positive, illustrating that fatigue predicted FCR.

**TABLE 3:** Tests of mediation of fatigue by fear of cancer recurrence (FCR) in predicting psychological Distress

		Effect of fatigue on FCR (*a* path)	Effect of FCR on distress (*b* path)	Total effect (*c* path)	Direct effect (*c’* path)	Indirect effect (*ab* path)			
	Model summary	*B*	*B*	*B*	*B*	*β*	95% LLCI	95% ULCI	Effect size (*ab/c*’)
Depression	*R* = 0.47	0.11**	1.04	0.75**	0.63**	0.12	−0.04	0.32	0.19
	*R* ^2^ = 0.22								
	*F* (1, 90) = 25.69**								
Anxiety	*R* = 0.39	0.11**	0.98*	0.51**	0.40**	0.11	**0.003**	**0.22**	0.27
	*R* ^2^ = 0.16								
	*F* (1, 90) = 16.57**								
Stress	*R* = 0.48	0.11**	1.55*	0.78**	0.60**	0.18	**0.01**	**0.39**	0.29
	*R* ^2^ = 0.23								
	*F* (1, 90) = 27.56**								

*Note*: **Bold** indicates significant indirect effects.

Abbreviations: FCR, fear of cancer recurrence; LLCI, lower limit confidence interval; ULCI, upper limit confidence intervals.

*p < 0.05; **p < 0.01 (two‐tailed).

**FIGURE 2:**
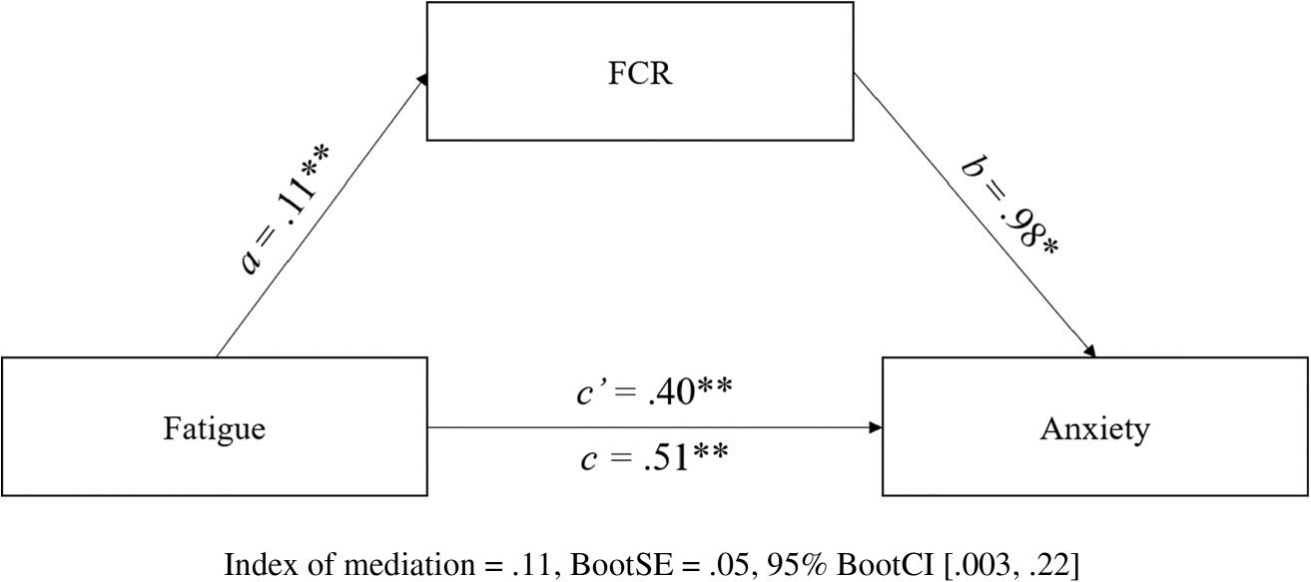
Partial mediation of fatigue by fear of cancer recurrence (FCR) in predicting anxiety. Figure 2 depicts the mediation model tested using Model 4 of Hayes’ PROCESS in predicting anxiety. *p < 0.05; **p < 0.01 level (two‐tailed)

The indirect effect of fatigue via FCR (*ab*) was significant and positive for anxiety (Figure 2). Here, the total effect of fatigue on anxiety (*c* path) reduced from *B* = 0.51 to *B* = 0.40 and remained significant (*p* < 0.001). These results demonstrate partial mediation of fatigue by FCR, in which fatigue both directly predicted anxiety and indirectly predicted anxiety via FCR. The effect size for predicting anxiety indicated the indirect effect was 27% of the respective direct effect of fatigue on anxiety.

**FIGURE 3:**
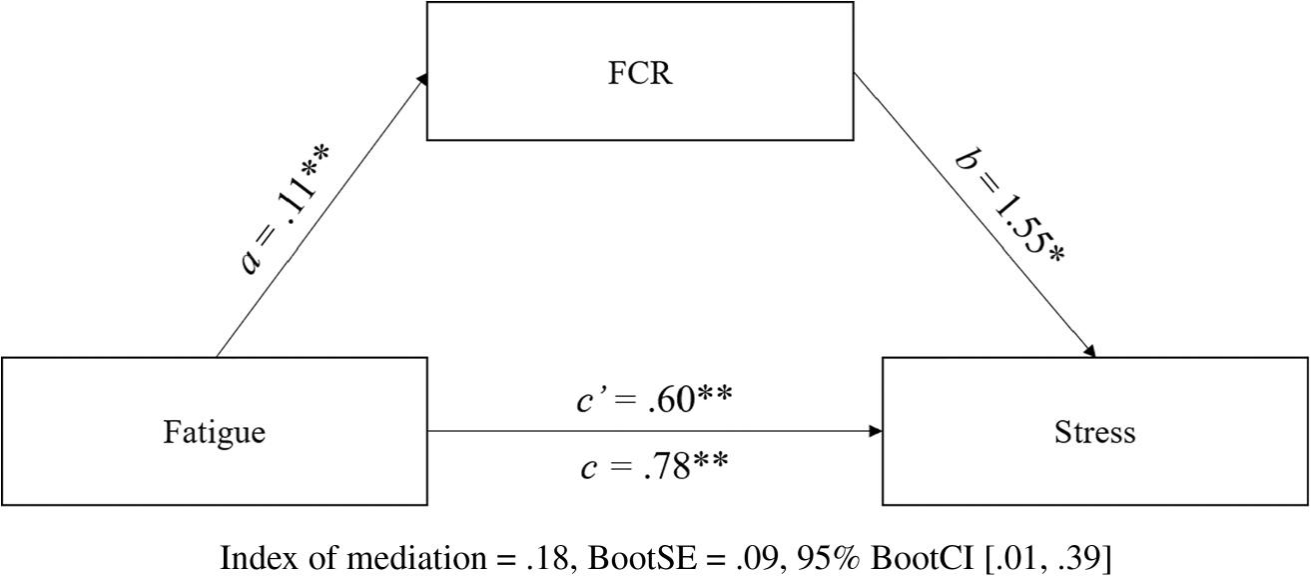
Partial mediation of fatigue by fear of cancer recurrence (FCR) in predicting stress. Figure 3 depicts the mediation model tested using Model 4 of Hayes’ PROCESS in predicting stress. *p < 0.05; **p < 0.01 level (two‐tailed)

The indirect effect of fatigue via FCR (*ab*) for stress was also significant and positive (Figure 3). The total effect of fatigue on stress reduced from *B* = 0.78 to *B* = 0.60 and remained significant (*p* < 0.001). These results indicate partial mediation of fatigue by FCR in predicting stress, in which fatigue both directly predicted stress and indirectly predicted stress via FCR. The effect size for predicting stress indicated the indirect effect was 29% of the respective direct effect of fatigue on stress.

**FIGURE 4:**
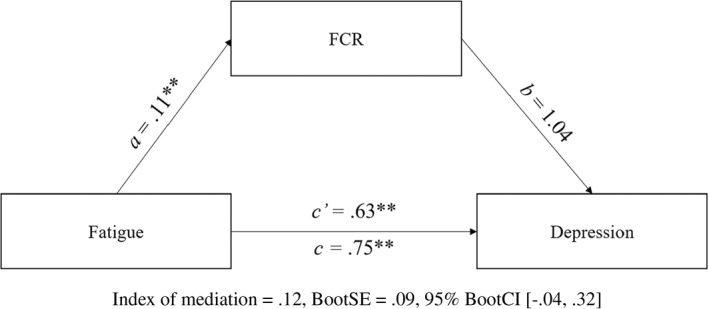
No mediation of fatigue by fear of cancer recurrence (FCR) in predicting depression. Figure 4 depicts the mediation model tested using Model 4 of Hayes’ PROCESS in predicting depression, in which no mediation of fatigue by FCR was found in predicting depression. *p < 0.05; **p < 0.01 level (two‐tailed)

For depression, the CI for the indirect effect of fatigue via FCR (*ab*) spanned zero and therefore the criteria for mediation was not met (Figure 4). Whilst FCR and depression were positively correlated (*r* = 0.35, *p* < 0.01), FCR did not significantly predict depression in the mediation model (b path; *B* = 1.04, *p* = 0.09). However, the total effect of fatigue on depression was significant (*B* = 0.75, *p* < 0.001), demonstrating that fatigue directly and positively predicts depression.

## 4 DISCUSSION

Our findings provided new insight into the mediating role of FCR in the link between fatigue and psychological distress in mothers who are breast cancer survivors. Our results indicate fatigue directly predicts depression in mothers who are breast cancer survivors, not necessarily via FCR as expected. However, FCR did mediate the relationships between fatigue and anxiety and fatigue and stress in accordance with our proposed model. More specifically, higher levels of fatigue predicted higher levels of anxiety and stress directly, and indirectly through higher levels of FCR. The mediating role of FCR demonstrated here suggests that the pathway from fatigue to anxiety and stress occurs in two steps: 1) fatigue triggers FCR; and 2) FCR, in turn, is associated with symptoms of anxiety and stress. The current study therefore contributes an important perspective in examining FCR; as a potential pathway to distress in response to ongoing fatigue among mothers who are breast cancer survivors, and as a potential mechanism of action for an intervention to reduce their psychological distress.

Our findings also provide further support for the relationship between symptom burden associated with cancer‐related fatigue and cancer survivors' fears about their cancer recurring (Arès et al., 2014; Crist & Grunfeld, 2013; Kelada et al., 2019; Koch et al., 2013; Lebel et al., 2018; Simard et al., 2013; Vickberg, 2003). In line with Leventhal's CSM and Mishel's Uncertainty in Illness theories (Lebel et al., 2018; Lee‐Jones et al., 1997; Mishel, 1988), the unpredictable and uncertain nature of cancer may lead to heightened sensitivity towards benign somatic experiences, such as fatigue, so that physical symptoms may be interpreted as cancer recurrence (Koch et al., 2013; Lebel et al., 2014, 2018; Lee‐Jones et al., 1997; McGinty et al., 2016; Mishel, 1988; Simard et al., 2013). Increased vigilance to physical symptoms can also lead to false positives, the identification of a symptom that is unrelated to the disease (Lee‐Jones et al., 1997), which subsequently trigger anxious preoccupation and rumination about cancer recurring (Lebel et al., 2018; Lee‐Jones et al., 1997; Vickberg, 2003).

Notably, higher levels of fatigue also directly predicted higher levels of anxiety and stress in our study, irrespective of mothers' fear about their cancer recurring. This may be attributed to the additional challenges that mothers often endure due to fatigue. For instance, fatigue may significantly interfere with mothers' day‐to‐day activities in fulfiling parenting responsibilities, which subsequently threatens their sense of identity that is tightly intertwined with their role as a parent (Kuswanto et al., 2018; Mackenzie, 2014; Tavares et al., 2017). Furthermore, fatigue may necessitate that mothers rely more on family and friends for practical support with parenting and daily house chores (Coyne & Borbasi, 2007; Elmberger et al., 2005; Fisher & O’Connor, 2012; Fitch et al., 1999; Kim et al., 2012; Öhlén & Holm, 2006), though some mothers may have difficulty trusting others to care for their children (Bell & Ristovski‐Slijepcevic, 2011; Billhult & Segesten, 2003; Elmberger et al., 2000; Kim et al., 2012). Past studies have indicated that mothers perceived societal demands to continue taking responsibility for child care and housekeeping, despite their physical limitations after receiving a cancer diagnosis (Fisher & O’Connor, 2012; Kuswanto et al., 2018; Semple & McCance, 2010). Thus, mothers who continue to experience fatigue are likely to feel anxious and worry about their ability to care for their children, suggesting an important relationship between fatigue and parenting efficacy in mothers who are breast cancer survivors. Whilst future research could elucidate this further, it is plausible that fatigue is a detrimental factor that distinguishes a mother's ability to fulfil their parenting roles before and after their cancer diagnosis, and that poor parenting efficacy might also mediate the relationship between fatigue and symptoms of anxiety or stress.

Contrary to our hypothesis, FCR did not mediate the relationship between fatigue and symptoms of depression; rather, more fatigue was directly associated with more symptoms of depression. Past research has suggested that fatigue may serve as a constant reminder of the cancer and its associated challenges in juggling between caring for their own needs and meeting their children's needs, which may lead to the rumination about the sense of loss, sadness and guilt for being the perceived less‐than‐ideal mother (Fisher & O’Connor, 2012; Kuswanto et al., 2018; Tavares et al., 2017). However, we are cautious in making predictive interpretations given fatigue is a hallmark of depressive symptoms (American Psychiatric Association, 2013). Whilst the DASS21 scale does not directly assess for the severity of fatigue within the depression domain, mothers may have reported depressive symptoms such as anhedonia, feeling low and pessimistic or lacking in initiative because of fatigue; likewise, mothers might report higher fatigue stemming from their depressive symptoms (Lovibond, 1998; Lovibond & Lovibond, 1995; Yavuzsen et al., 2009).

### 4.1 Clinical implications

Our findings highlight the need for ongoing assessment of mothers' physical and psychological wellbeing, as well as their fears about their cancer recurring. The cognitive processing system in FCR, which involves misinterpretation of fatigue and other physical symptoms (Fardell et al., 2016), indicates that psycho‐education and cognitive‐based therapy is suitable in reducing distress (Lee‐Jones et al., 1997). For instance, Lengacher et al. (2014) reported a short‐term Mindfulness‐based Stress Reduction in Breast Cancer (MBSR(BC)) programme was effective in reducing FCR and improving physical functioning post‐treatment in breast cancer survivors, resulting in reduced symptoms of stress and anxiety. Mindfulness‐based therapies that assist self‐regulation through practicing non‐judgemental acceptance of somatic and emotional experiences, and focussing on the present moment rather than reacting or ruminating, may therefore be beneficial for mothers who are breast cancer survivors by addressing the preoccupation and ruminating thinking patterns observed in FCR (Lengacher et al., 2014). A recent systematic review and meta‐analysis also demonstrated the effectiveness of contemporary CBT‐based therapies in reducing FCR by targeting how cancer survivors engage with the thoughts about recurrence, such as focussing on the processes of cognition (e.g., worry, rumination and attentional biases) rather than the content of it (Tauber et al., 2019). Physical activity‐based interventions may also be helpful in addressing fatigue, depression and anxiety in mothers who are breast cancer survivors. Breast cancer survivors who have been more physically active have reported experiencing lower fatigue and depressive symptomatology when compared to those who have been less physically active (Aguiñaga et al., 2018). Another study has also examined the efficacy of a combination of social‐cognitive therapy and physical activity behaviour change intervention (Rogers et al., 2017). The Better Exercise Adherence after Treatment for Cancer (BEAT Cancer) intervention, involving supervised exercise sessions, exercise monitoring and face‐to‐face counselling sessions was efficacious in reducing fatigue, depressive symptomatology and anxiety up to 3 months post‐primary treatment for breast cancer. In addition, a recent study showed physical activity may alleviate FCR in cancer survivors by means of increasing sense of control over their health, decreasing perceived chronicity of cancer and mitigating cancer implications (Séguin Leclair et al., 2021). Such findings underscore the value of engaging in regular physical activity for breast cancer survivors.

Mothers, in particular, may present with additional challenges due to their parenting responsibilities and the impact of cancer recurring on their children's and family's wellbeing. A recent systematic review by Kuswanto et al. (2018) reported mothers may experience disruption to self‐identity and parenting confidence, and that their cancer diagnosis has considerable impact on their children's emotions, which may include becoming distressed, frightened, worried and being avoidant and withdrawn. Shedding light on the relationships between fatigue, FCR and psychological distress highlights the importance of providing ongoing parenting support for both mothers and their families. This could be in a form of instrumental (e.g., cooking, housecleaning, and caring for their children), emotional (e.g., counselling for the family or for their children), or social (e.g., having a support group with other mothers who are breast cancer survivors) support for families affected by breast cancer (Fitch et al., 1999).

Previous studies emphasised that many breast cancer survivors have unmet information needs, particularly with regards to the long‐term physical, psychological and social sequelae of cancer and the availability of support, which can have a significant impact on their FCR and psychological distress (Cheng et al., 2014; Connell et al., 2006; Vivar & McQueen, 2005). Kelada et al. (2019) demonstrated adult survivors of childhood cancer with unmet information needs for managing pain and fatigue were more likely to experience higher FCR than those with no information needs. This is likely attributable to rumination on the possibility of cancer recurrence when cancer survivors are unable to contextualise their experience of physical symptoms (Kelada et al., 2019), which can exacerbate their FCR (Kelada et al., 2019; Lee‐Jones et al., 1997; McGinty et al., 2016; Vickberg, 2003). These findings highlight the need for ongoing healthcare support in providing cancer‐related information and monitoring for physical and psychological symptoms during long‐term follow‐ups in order to manage potential FCR and be informed about how to communicate effectively to their family members with regards to their long‐term survivorship care. The contribution of unmet information needs to higher FCR is also identified in the cognitive processing model of FCR (Fardell et al., 2016), and future research should incorporate unmet information needs as a predictor of FCR.

### 4.2 Limitations and directions for future research

The current study involved a small sample size, which increases the potential for Type II errors and restricts statistical power in our analyses. Also, past studies have shown that anxiety and hyper‐sensitivity towards internal triggers can also be perpetuated by external triggers that may serve as constant reminders of cancer, such as medical appointments, the anniversary date of the cancer diagnosis, and conversations about cancer with friends or through the media (McGinty et al., 2016). Thus, we did not exclude the possibility that the administration of measures regarding participants' level of fatigue and FCR may lead to an overestimation of their typical psychological distress, particularly their anxiety level.

Most of the participants were also older and employed, currently in a relationship, and had attained a tertiary education. Understanding that age, relationship status, socioeconomic status and cancer characteristics may influence the levels of fatigue, FCR and psychological distress (Crist & Grunfeld, 2013; Gotze et al., 2015; Koch et al., 2014; Kuswanto et al., 2018; Mehnert et al., 2009), further research is required to examine whether our findings can be generalised to other mothers with different socio‐economic backgrounds and cancer diagnoses. Furthermore, due to the cross‐sectional nature of our study, the assumed casual pathway in which fatigue influences psychological distress via FCR would need to be further investigated via a longitudinal study design.

Looking ahead, future research examining this model in which FCR represents a pathway from fatigue to psychological distress should also explore the nature of mothers' fears about their cancer recurring, in addition to FCR severity. Vickberg (2003) explains there are varying aspects of women's FCR, including fears about the possibilities of death, health deterioration and further treatment, emotional difficulties and physical limitations, and threats to identity and womanhood, body image and sexuality, and their roles and relationships. These fears have also correlated with psychological distress, intrusive thoughts and avoidance (Vickberg, 2003). Better understanding of the nature of mothers' FCR would have clinical benefits, as knowing the nature of their fears is necessary in addressing their specific concerns about cancer recurrence. Furthermore, it would be meaningful for future research to test other models in which the direction of the pathway is explored; for instance, whether psychological distress predicts fatigue mediated by FCR, FCR predicts psychological distress mediated by fatigue, or FCR predicts fatigue mediated by psychological distress. Additionally, future research could further examine if the pathways demonstrated in the model also occur in sample of women who are not mothers, as it may shed light into how fatigue interrupts womanhood and other daily functioning beyond the mothering role.

## 5 CONCLUSIONS

Our findings indicate that FCR provides a pathway from cancer‐related fatigue to anxiety and stress in mothers who are breast cancer survivors, where higher levels of fatigue predict higher levels of anxiety and stress directly, and indirectly through higher levels of FCR. Furthermore, cancer‐related fatigue predicts depression in mothers who are breast cancer survivors. Better understanding of the role of cancer‐related fatigue and FCR can have clinical benefits in providing better psychosocial support tailored to their specific needs, particularly if they continue to struggle with psychological distress. We recommend that future research extends this model in which FCR represents a pathway from fatigue to psychological distress by also considering the nature of mothers' fears about their cancer recurring and their unmet information needs.

## AUTHOR CONTRIBUTIONS

**Carissa Nadia Kuswanto:** Conceptualization; Investigation; Data curation; Formal analysis; Methodology; Writing – original draft. **Jessica Sharp:** Supervision; Formal analysis; Writing – review and editing. **Lesley Stafford:** Supervision; Writing – review and editing. **Penelope Schofield:** Supervision; Writing – review and editing.

## CONFLICT OF INTEREST

The author declares that there is no conflict of interest that could be perceived as prejudicing the impartiality of the research reported.

## Data Availability

The data that support the findings of this study are available from the corresponding author upon reasonable request.
